# 2-Amino-6-methyl­pyridinium 2-carb­oxy­benzoate

**DOI:** 10.1107/S1600536811033174

**Published:** 2011-08-27

**Authors:** Madhukar Hemamalini, Ibrahim Abdul Razak, Hoong-Kun Fun

**Affiliations:** a X-ray Crystallography Unit, School of Physics, Universiti Sains Malaysia, 11800 USM, Penang, Malaysia

## Abstract

In the title mol­ecular salt, C_6_H_9_N_2_^+^·C_8_H_5_O_4_^−^, an intra­molecular O—H⋯O hydrogen bond occurs within the anion, thereby generating an *S*(7) ring, which may correlate with the fact that both the carb­oxy­lic acid and carboxyl­ate groups are almost coplanar with their attached rings [dihedral angles = 2.9 (3) and 5.2 (3)°, respectively]. In the crystal, each cation is linked to its adjacent anion by two N—H⋯O hydrogen bonds; the dihedral angle between the pyridine and benzene rings is 2.22 (10)°. The ion pairs are linked by further N—H⋯O inter­actions.

## Related literature

For related structures, see: Navarro Ranninger *et al.* (1985 ▶); Luque *et al.* (1997 ▶); Jin *et al.* (2000 ▶); Schuckmann *et al.* (1978 ▶); Küppers *et al.* (1985 ▶); Jessen (1990 ▶); Hemamalini & Fun (2010**a* ▶,b* ▶); Quah *et al.* (2010 ▶). For hydrogen-bond motifs, see: Bernstein *et al.* (1995 ▶).
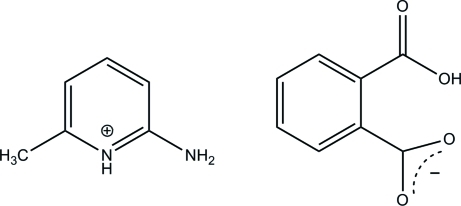


## Experimental

### 

#### Crystal data

C_6_H_9_N_2_^+^·C_8_H_5_O_4_^−^
*M* *_r_* = 274.27
Triclinic, 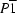

*a* = 7.473 (2) Å
*b* = 8.386 (3) Å
*c* = 11.818 (4) Å
α = 97.401 (6)°
β = 102.940 (7)°
γ = 109.616 (6)°
*V* = 662.9 (4) Å^3^
*Z* = 2
Mo *K*α radiation
μ = 0.10 mm^−1^
*T* = 296 K
1.00 × 0.20 × 0.10 mm

#### Data collection

Bruker APEXII DUO CCD diffractometer
Absorption correction: multi-scan (*SADABS*; Bruker, 2009 ▶) *T*_min_ = 0.905, *T*_max_ = 0.990
12166 measured reflections
3706 independent reflections
2219 reflections with *I* > 2σ(*I*)
*R*_int_ = 0.044

#### Refinement

*R*[*F* ^2^ > 2σ(*F* ^2^)] = 0.055
*wR*(*F* ^2^) = 0.190
*S* = 1.05
3706 reflections
194 parameters
H atoms treated by a mixture of independent and constrained refinement
Δρ_max_ = 0.20 e Å^−3^
Δρ_min_ = −0.24 e Å^−3^

### 

Data collection: *APEX2* (Bruker, 2009 ▶); cell refinement: *SAINT* (Bruker, 2009 ▶); data reduction: *SAINT*; program(s) used to solve structure: *SHELXTL* (Sheldrick, 2008 ▶); program(s) used to refine structure: *SHELXTL*; molecular graphics: *SHELXTL*; software used to prepare material for publication: *SHELXTL* and *PLATON* (Spek, 2009 ▶).

## Supplementary Material

Crystal structure: contains datablock(s) global, I. DOI: 10.1107/S1600536811033174/hb6359sup1.cif

Structure factors: contains datablock(s) I. DOI: 10.1107/S1600536811033174/hb6359Isup2.hkl

Supplementary material file. DOI: 10.1107/S1600536811033174/hb6359Isup3.cml

Additional supplementary materials:  crystallographic information; 3D view; checkCIF report

## Figures and Tables

**Table 1:** Hydrogen-bond geometry (Å, °)

*D*—H⋯*A*	*D*—H	H⋯*A*	*D*⋯*A*	*D*—H⋯*A*
O1—H1*O*1⋯O3	0.84	1.55	2.393 (2)	174
N1—H1*N*1⋯O4	0.98 (2)	1.71 (2)	2.692 (2)	175 (2)
N2—H1*N*2⋯O2^i^	0.96 (3)	1.99 (2)	2.940 (3)	172 (2)
N2—H2*N*2⋯O3	0.92 (2)	2.04 (2)	2.936 (3)	165 (2)

Symmetry code: (i) 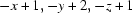.

**Fig. 1.**
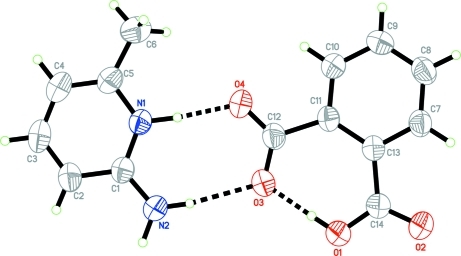
The asymmetric unit of the title compound, showing 50% probability displacement ellipsoids. Intramolecular hydrogen bonds shown by dashed lines.

**Fig. 2.**
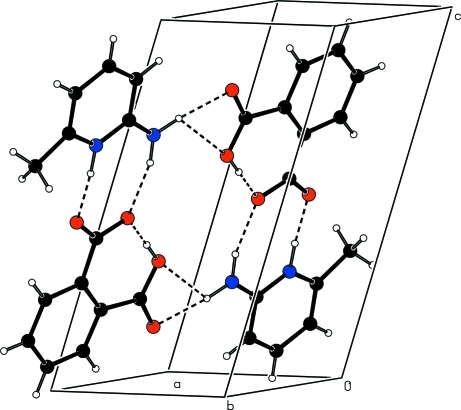
The crystal packing of title compound (I) showing a dimer. Dashed lines represents hydrogen bonding.

## supplementary crystallographic information

### Comment

There are numerous examples of 2-amino-substituted pyridine compounds in which the 2-aminopyridines act as ligands (Navarro Ranninger *et al.*, 1985) or as protonated cations (Luque *et al.*, 1997; Jin *et al.*, 2000). Phthalic acid forms hydrogenphthalate salts with various organic and other compounds. The crystal structures of hydrogenphthalates include calcium phthalate monohydrate (Schuckmann *et al.*, 1978), lithium hydrogen phthalate monohydrate (Küppers *et al.*, 1985) and tetramethylammonium hydrogen phthalate (Jessen, 1990) which have been reported in the literature. Recently, we have reported the crystal structures of 2-amino-5-chloro pyridinium 2-carboxybenzoate-benzene-1,2-dicarboxylic acid (Hemamalini & Fun, 2010*a*), 2-amino-5-bromopridinium 2-carboxybenzoate (Quah *et al.*, 2010) and 2-amino-5-methylpyridinium 2-carboxybenzoate (Hemamalini & Fun, 2010*b*) from our laboratory. In a continuation of our studies of pyridinium derivatives, the crystal structure determination of the title compound (I) has been undertaken.

In the title salt, (I), the asymmetric unit contains a protonated 2-amino-6-methylpyridinium cation and a hydrogenphthalate anion as shown in Fig.1.  In the 2-amino-6-methylpyridinium cation, a wider than normal angle [C1—N1—C5 = 123.64 (16)°] is subtended at the protonated N1 atom. The pyridine ring is essentially planar, with a maximum deviation of 0.007 (2) Å for atom C2.  The dihedral angle between the pyridine (N1/C1–C5) and benzene (C7–C11/C13) rings is 2.22 (10)°.

In the crystal structure (Fig. 2), the cations and anions are connected *via* intermolecular N—H···O and intramolecular O—H···O (Table 1) hydrogen bonds forming dimers.  These dimers contain *R*^2^_2_(8), *R*^1^_2_(4) and *S*(7) ring motifs.

### Experimental

A hot methanol solution (20 ml) of 2-amino-6-methylpyridine (54 mg, Aldrich) and phthalic acid (41 mg, Merck) were mixed and warmed over a heating magnetic stirrer hotplate for a few minutes. The resulting solution was allowed to cool slowly at room temperature and colourless needles of the title compound appeared after a few days.

### Refinement

Atoms H1N1, H1N2 and H2N2 were located from difference Fourier maps and refined freely [N–H = 0.92 (3)–0.99 (3) Å]. The remaining H atoms were positioned geometrically [C–H = 0.93–0.96 Å and O–H = 0.8434 Å] and were refined using a riding model, with *U*_iso_(H) = 1.2 or 1.5 *U*_eq_(C). A rotating group model was used for the methyl group.

### Crystal data

**Table:**

C_6_H_9_N_2_^+^·C_8_H_5_O_4_^−^	*Z* = 2
*M**_r_* = 274.27	*F*(000) = 288
Triclinic, *P*1	*D*_x_ = 1.374 Mg m^−^^3^
Hall symbol: -P 1	Mo *K*α radiation, λ = 0.71073 Å
*a* = 7.473 (2) Å	Cell parameters from 3645 reflections
*b* = 8.386 (3) Å	θ = 2.6–27.4°
*c* = 11.818 (4) Å	µ = 0.10 mm^−^^1^
α = 97.401 (6)°	*T* = 296 K
β = 102.940 (7)°	Needle, colourless
γ = 109.616 (6)°	1.00 × 0.20 × 0.10 mm
*V* = 662.9 (4) Å^3^	

### Data collection

**Table:**

Bruker APEXII DUO CCD diffractometer	3706 independent reflections
Radiation source: fine-focus sealed tube	2219 reflections with *I* > 2σ(*I*)
graphite	*R*_int_ = 0.044
φ and ω scans	θ_max_ = 29.9°, θ_min_ = 1.8°
Absorption correction: multi-scan (*SADABS*; Bruker, 2009)	*h* = −10→10
*T*_min_ = 0.905, *T*_max_ = 0.990	*k* = −11→11
12166 measured reflections	*l* = −16→16

### Refinement

**Table:**

Refinement on *F*^2^	Primary atom site location: structure-invariant direct methods
Least-squares matrix: full	Secondary atom site location: difference Fourier map
*R*[*F*^2^ > 2σ(*F*^2^)] = 0.055	Hydrogen site location: inferred from neighbouring sites
*wR*(*F*^2^) = 0.190	H atoms treated by a mixture of independent and constrained refinement
*S* = 1.05	*w* = 1/[σ^2^(*F*_o_^2^) + (0.1052*P*)^2^ + 0.0321*P*] where *P* = (*F*_o_^2^ + 2*F*_c_^2^)/3
3706 reflections	(Δ/σ)_max_ < 0.001
194 parameters	Δρ_max_ = 0.20 e Å^−^^3^
0 restraints	Δρ_min_ = −0.24 e Å^−^^3^

### Special details

**Table:**

Geometry. All esds (except the esd in the dihedral angle between two l.s. planes) are estimated using the full covariance matrix. The cell esds are taken into account individually in the estimation of esds in distances, angles and torsion angles; correlations between esds in cell parameters are only used when they are defined by crystal symmetry. An approximate (isotropic) treatment of cell esds is used for estimating esds involving l.s. planes.
Refinement. Refinement of F^2^ against ALL reflections. The weighted R-factor wR and goodness of fit S are based on F^2^, conventional R-factors R are based on F, with F set to zero for negative F^2^. The threshold expression of F^2^ > 2sigma(F^2^) is used only for calculating R-factors(gt) etc. and is not relevant to the choice of reflections for refinement. R-factors based on F^2^ are statistically about twice as large as those based on F, and R- factors based on ALL data will be even larger.

### Fractional atomic coordinates and isotropic or equivalent isotropic displacement parameters (Å^2^)

**Table:**

	*x*	*y*	*z*	*U*_iso_*/*U*_eq_	
O1	0.3997 (3)	0.99186 (18)	0.64341 (12)	0.0720 (5)	
H1O1	0.3652	0.8964	0.5951	0.108*	
O2	0.4150 (3)	1.09584 (17)	0.82518 (13)	0.0727 (5)	
O3	0.3231 (2)	0.71890 (18)	0.51427 (12)	0.0682 (4)	
O4	0.2170 (2)	0.44177 (18)	0.51346 (12)	0.0694 (4)	
N1	0.2619 (2)	0.33871 (18)	0.29978 (13)	0.0452 (3)	
N2	0.3912 (3)	0.6236 (2)	0.28428 (16)	0.0562 (4)	
C1	0.3333 (2)	0.4560 (2)	0.23665 (14)	0.0441 (4)	
C2	0.3430 (3)	0.3937 (2)	0.12220 (15)	0.0504 (4)	
H2A	0.3931	0.4707	0.0766	0.060*	
C3	0.2783 (3)	0.2199 (3)	0.07971 (17)	0.0574 (5)	
H3A	0.2823	0.1782	0.0039	0.069*	
C4	0.2057 (3)	0.1023 (3)	0.14768 (18)	0.0584 (5)	
H4A	0.1623	−0.0164	0.1174	0.070*	
C5	0.1989 (3)	0.1630 (2)	0.25911 (17)	0.0503 (4)	
C6	0.1328 (4)	0.0531 (3)	0.3429 (2)	0.0694 (6)	
H6A	0.0575	−0.0647	0.2996	0.104*	
H6B	0.0516	0.0958	0.3808	0.104*	
H6C	0.2465	0.0575	0.4023	0.104*	
C7	0.2521 (3)	0.7860 (2)	0.87715 (15)	0.0481 (4)	
H7A	0.2875	0.8912	0.9299	0.058*	
C8	0.1716 (3)	0.6332 (3)	0.91302 (16)	0.0539 (4)	
H8A	0.1541	0.6364	0.9886	0.065*	
C9	0.1182 (3)	0.4775 (3)	0.83617 (17)	0.0561 (5)	
H9A	0.0626	0.3742	0.8589	0.067*	
C10	0.1472 (3)	0.4744 (2)	0.72467 (16)	0.0511 (4)	
H10A	0.1106	0.3678	0.6733	0.061*	
C11	0.2297 (2)	0.6265 (2)	0.68645 (13)	0.0419 (4)	
C12	0.2586 (3)	0.5937 (2)	0.56344 (15)	0.0481 (4)	
C13	0.2820 (2)	0.7880 (2)	0.76533 (14)	0.0424 (4)	
C14	0.3712 (3)	0.9700 (2)	0.74459 (16)	0.0498 (4)	
H1N1	0.251 (3)	0.383 (3)	0.378 (2)	0.068 (6)*	
H1N2	0.454 (4)	0.707 (3)	0.243 (2)	0.075 (7)*	
H2N2	0.394 (3)	0.663 (3)	0.361 (2)	0.074 (7)*	

### Atomic displacement parameters (Å^2^)

**Table:**

	*U*^11^	*U*^22^	*U*^33^	*U*^12^	*U*^13^	*U*^23^
O1	0.1160 (13)	0.0466 (8)	0.0533 (8)	0.0208 (7)	0.0380 (8)	0.0130 (6)
O2	0.1115 (13)	0.0458 (8)	0.0560 (8)	0.0212 (8)	0.0330 (8)	0.0025 (6)
O3	0.0998 (11)	0.0543 (8)	0.0468 (8)	0.0175 (7)	0.0337 (7)	0.0086 (6)
O4	0.1065 (12)	0.0497 (8)	0.0519 (8)	0.0250 (8)	0.0338 (8)	0.0029 (6)
N1	0.0491 (8)	0.0417 (8)	0.0408 (7)	0.0147 (6)	0.0123 (6)	0.0028 (6)
N2	0.0740 (10)	0.0417 (8)	0.0501 (9)	0.0160 (7)	0.0239 (8)	0.0053 (7)
C1	0.0449 (8)	0.0456 (9)	0.0404 (8)	0.0185 (7)	0.0096 (6)	0.0052 (7)
C2	0.0558 (10)	0.0596 (11)	0.0411 (9)	0.0285 (8)	0.0149 (7)	0.0089 (8)
C3	0.0618 (11)	0.0663 (12)	0.0447 (9)	0.0328 (9)	0.0114 (8)	−0.0023 (8)
C4	0.0616 (11)	0.0504 (10)	0.0579 (11)	0.0235 (8)	0.0119 (9)	−0.0037 (8)
C5	0.0488 (9)	0.0428 (9)	0.0540 (10)	0.0153 (7)	0.0118 (7)	0.0038 (7)
C6	0.0795 (14)	0.0504 (11)	0.0794 (15)	0.0201 (10)	0.0301 (11)	0.0171 (10)
C7	0.0554 (10)	0.0511 (10)	0.0409 (9)	0.0231 (8)	0.0168 (7)	0.0065 (7)
C8	0.0629 (11)	0.0627 (12)	0.0449 (9)	0.0269 (9)	0.0250 (8)	0.0165 (8)
C9	0.0638 (11)	0.0508 (10)	0.0538 (11)	0.0150 (8)	0.0236 (8)	0.0184 (8)
C10	0.0577 (10)	0.0441 (9)	0.0453 (9)	0.0130 (7)	0.0149 (7)	0.0065 (7)
C11	0.0411 (8)	0.0473 (9)	0.0355 (8)	0.0165 (6)	0.0096 (6)	0.0062 (6)
C12	0.0519 (9)	0.0511 (10)	0.0378 (8)	0.0168 (7)	0.0120 (7)	0.0063 (7)
C13	0.0442 (8)	0.0451 (9)	0.0382 (8)	0.0183 (7)	0.0114 (6)	0.0072 (7)
C14	0.0596 (10)	0.0451 (9)	0.0452 (9)	0.0190 (7)	0.0171 (7)	0.0097 (7)

### Geometric parameters (Å, °)

**Table:**

O1—C14	1.286 (2)	C4—H4A	0.9300
O1—H1O1	0.8434	C5—C6	1.491 (3)
O2—C14	1.223 (2)	C6—H6A	0.9600
O3—C12	1.271 (2)	C6—H6B	0.9600
O4—C12	1.236 (2)	C6—H6C	0.9600
N1—C1	1.350 (2)	C7—C8	1.386 (3)
N1—C5	1.369 (2)	C7—C13	1.390 (2)
N1—H1N1	0.99 (3)	C7—H7A	0.9300
N2—C1	1.326 (2)	C8—C9	1.367 (3)
N2—H1N2	0.95 (3)	C8—H8A	0.9300
N2—H2N2	0.92 (3)	C9—C10	1.381 (3)
C1—C2	1.413 (2)	C9—H9A	0.9300
C2—C3	1.356 (3)	C10—C11	1.398 (2)
C2—H2A	0.9300	C10—H10A	0.9300
C3—C4	1.397 (3)	C11—C13	1.418 (2)
C3—H3A	0.9300	C11—C12	1.521 (2)
C4—C5	1.368 (3)	C13—C14	1.524 (3)
			
C14—O1—H1O1	111.8	H6A—C6—H6C	109.5
C1—N1—C5	123.64 (16)	H6B—C6—H6C	109.5
C1—N1—H1N1	117.4 (13)	C8—C7—C13	122.48 (17)
C5—N1—H1N1	118.9 (13)	C8—C7—H7A	118.8
C1—N2—H1N2	119.1 (15)	C13—C7—H7A	118.8
C1—N2—H2N2	122.3 (15)	C9—C8—C7	119.37 (17)
H1N2—N2—H2N2	118 (2)	C9—C8—H8A	120.3
N2—C1—N1	118.97 (16)	C7—C8—H8A	120.3
N2—C1—C2	122.93 (17)	C8—C9—C10	119.76 (17)
N1—C1—C2	118.09 (15)	C8—C9—H9A	120.1
C3—C2—C1	119.05 (18)	C10—C9—H9A	120.1
C3—C2—H2A	120.5	C9—C10—C11	122.13 (17)
C1—C2—H2A	120.5	C9—C10—H10A	118.9
C2—C3—C4	121.27 (18)	C11—C10—H10A	118.9
C2—C3—H3A	119.4	C10—C11—C13	118.15 (15)
C4—C3—H3A	119.4	C10—C11—C12	113.54 (15)
C5—C4—C3	119.58 (17)	C13—C11—C12	128.27 (15)
C5—C4—H4A	120.2	O4—C12—O3	121.50 (17)
C3—C4—H4A	120.2	O4—C12—C11	117.68 (15)
C4—C5—N1	118.35 (17)	O3—C12—C11	120.82 (16)
C4—C5—C6	125.27 (18)	C7—C13—C11	118.09 (15)
N1—C5—C6	116.36 (17)	C7—C13—C14	113.65 (15)
C5—C6—H6A	109.5	C11—C13—C14	128.25 (15)
C5—C6—H6B	109.5	O2—C14—O1	120.10 (17)
H6A—C6—H6B	109.5	O2—C14—C13	119.35 (16)
C5—C6—H6C	109.5	O1—C14—C13	120.55 (16)
			
C5—N1—C1—N2	−179.76 (16)	C10—C11—C12—O4	−3.4 (2)
C5—N1—C1—C2	0.1 (2)	C13—C11—C12—O4	174.11 (17)
N2—C1—C2—C3	−179.19 (17)	C10—C11—C12—O3	176.56 (16)
N1—C1—C2—C3	0.9 (2)	C13—C11—C12—O3	−5.9 (3)
C1—C2—C3—C4	−1.1 (3)	C8—C7—C13—C11	−0.8 (3)
C2—C3—C4—C5	0.2 (3)	C8—C7—C13—C14	179.70 (16)
C3—C4—C5—N1	0.8 (3)	C10—C11—C13—C7	1.3 (2)
C3—C4—C5—C6	−177.48 (18)	C12—C11—C13—C7	−176.07 (15)
C1—N1—C5—C4	−1.0 (3)	C10—C11—C13—C14	−179.27 (16)
C1—N1—C5—C6	177.44 (16)	C12—C11—C13—C14	3.3 (3)
C13—C7—C8—C9	−0.2 (3)	C7—C13—C14—O2	2.5 (3)
C7—C8—C9—C10	0.8 (3)	C11—C13—C14—O2	−176.88 (17)
C8—C9—C10—C11	−0.2 (3)	C7—C13—C14—O1	−177.23 (17)
C9—C10—C11—C13	−0.9 (3)	C11—C13—C14—O1	3.4 (3)
C9—C10—C11—C12	176.92 (17)		

### Hydrogen-bond geometry (Å, °)

**Table:**

*D*—H···*A*	*D*—H	H···*A*	*D*···*A*	*D*—H···*A*
O1—H1O1···O3	0.84	1.55	2.393 (2)	174
N1—H1N1···O4	0.98 (2)	1.71 (2)	2.692 (2)	175 (2)
N2—H1N2···O2^i^	0.96 (3)	1.99 (2)	2.940 (3)	172 (2)
N2—H2N2···O3	0.92 (2)	2.04 (2)	2.936 (3)	165 (2)

Symmetry codes: (i) −*x*+1, −*y*+2, −*z*+1.
